# Supplementary figures and images for: Feasibility of combining short tandem repeats (STRs) haplotyping with preimplantation genetic diagnosis (PGD) in screening for beta thalassemia
Source: PLoS One. 2022 Dec 7;17(12):e0278539. doi: 10.1371/journal.pone.0278539 (PMC9728894; doi:10.1371/journal.pone.0278539)

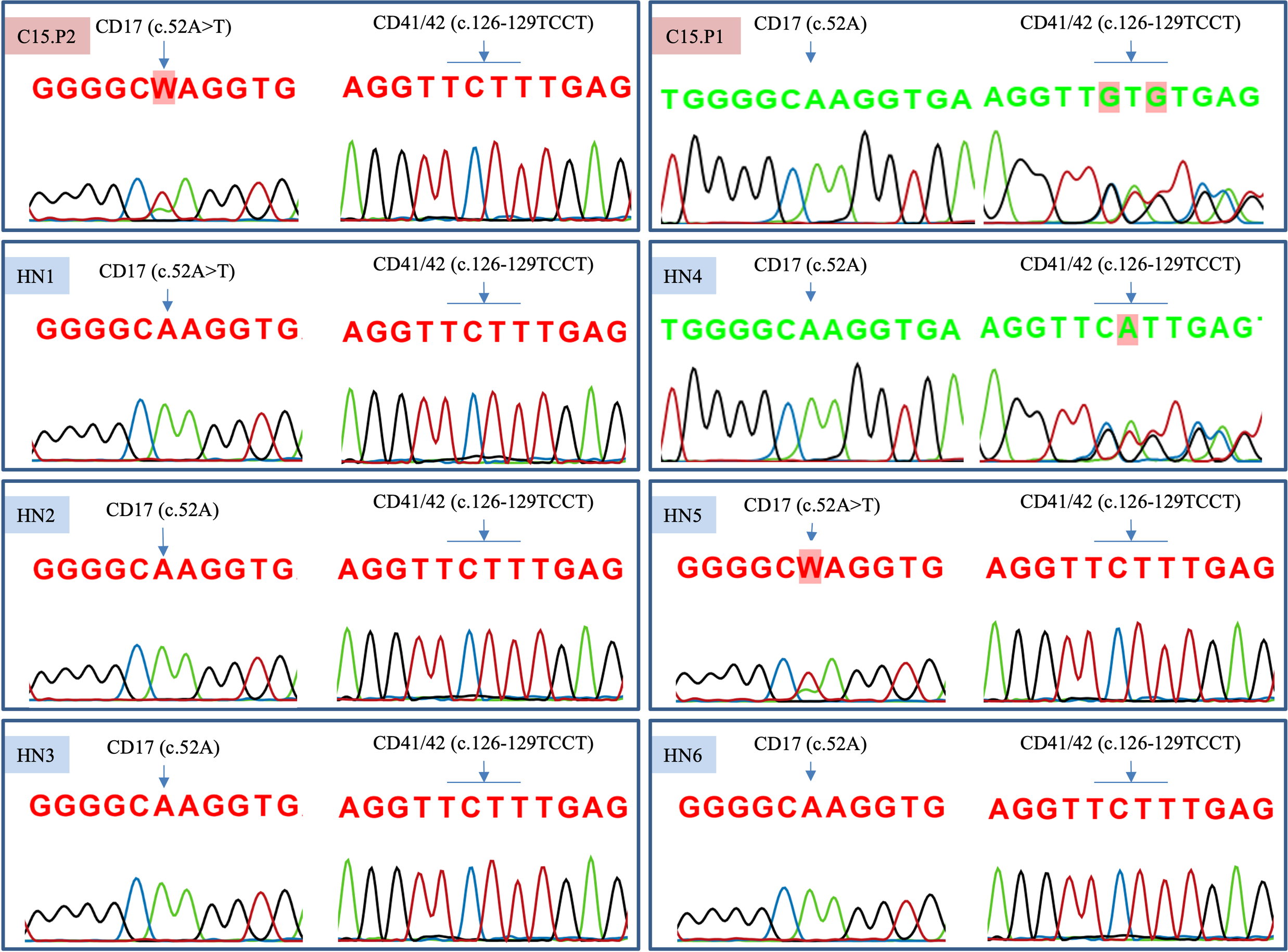

Supplement: S1 Fig — (TIFF) [file pone.0278539.s001.tiff]
