# Supplementary material for: Feasibility of combining short tandem repeats (STRs) haplotyping with preimplantation genetic diagnosis (PGD) in screening for beta thalassemia
Source: PLoS One. 2022 Dec 7;17(12):e0278539. doi: 10.1371/journal.pone.0278539 (PMC9728894; doi:10.1371/journal.pone.0278539)
Supplement: Attachment — Submitted filename: Comments.docx [file pone.0278539.s002.docx]

- The topic of this article lacks new ideas: PGD is a mature technology in the current field of reproductive medicine, and it is also a conventional indication for beta thalassemia. What is the novelty of this paper?
- Two STR markers named HBB5178, and D11S1760 have approximately close size range which can make misdiagnosis. It would be better to tag them with different fluorescent labels
- There are some grammatical. For instance:
  - Line119: " …family members were still used *for to increase* the…" should change into "to *increase*"
  - Line 151: "…3 over 88 failed…" should change into 3 out of 88 failed
  - Line 195: "…11 over 15 couples" should change into 11 out of 15 couples
- The discussion part lack the description of the experience from other research.
- The figures are blurred, it must be changed into a clear one.
