# Supplementary material for: Feasibility of combining short tandem repeats (STRs) haplotyping with preimplantation genetic diagnosis (PGD) in screening for beta thalassemia
Source: PLoS One. 2022 Dec 7;17(12):e0278539. doi: 10.1371/journal.pone.0278539 (PMC9728894; doi:10.1371/journal.pone.0278539)
Supplement: Attachment — Submitted filename: Response to Reviewers.docx [file pone.0278539.s003.docx]

RESPOND TO REVIEWER

**Submission Title:** Feasibility of combining short tandem repeats (STRs) haplotyping with preimplantation genetic diagnosis (PGD) in screening for beta thalassemia

Dear Editors and Reviewers,

We are grateful for your time reading and considering our work. Your feedbacks have helped us improved our manuscript substantially. We have thoroughly revised our work and, to the best of our ability as non-native speaker correct the grammar and syntax to meet with the publication standard. We hereby submit our revised manuscript together with detailed respond to each of the queries raised by the reviewer.

Respond to the Reviewer 1’s queries

**Q:** The topic of this article lacks new ideas: PGD is a mature technology in the current field of reproductive medicine, and it is also a conventional indication for beta thalassemia. What is the novelty of this paper?

**Answer:**

It is true that PGD is a mature technology in the current field of reproductive medicine and a conventional indication for beta thalassemia. Although PGD was first reported in the world in 1988, not until 2015, in a technology transfer project by Military Medical University, PGD was first introduced in Vietnam. Hence, with only 7 years of technological adaptation and optimization for Vietnamese population, there were many aspects that we can adjust and research on. Furthermore, not many hospitals in Vietnam have the adequate technological requirements as well as received the procedure of PGD for beta thalassemia. In this research, we would like to introduce the feasibility and reliability of this PGD strategy for beta thalassemia to other facilities in Vietnam and other developing countries with later obtention of the technology, thus, to reduce the rate of thalassemia carriers.

**Q:** Two STR markers named HBB5178, and D11S1760 have approximately close size range which can make misdiagnosis. It would be better to tag them with different fluorescent labels

**Answer:**

We will definitely change the fluorescence colour for the markers HBB5178, and D11S1760 in the next experiments. In this research, due to data in previous research, as well as our experience, the two markers are distinguishable with no overlaps. Furthermore, we used other markers to cross-check the results as at least three markers would confirm the presence of the allele.

**Q:** There are some grammatical errors

**Answer:** We have made appropriate corrections of the errors.

**Q:** The discussion part lacks the description of the experience from other research.

**Answer:** We have added some description of experience from studies in the same field.

**Q:** The figures are blurred, it must be changed into a clear one.

**Answer:** We have changed the figure and increase the resolution to 300 dpi.

Respond to the Reviewer 2’s queries

**Q:** If you are using some abbreviations, please give the complete name or description at the first place it is used in the article.

**Answer:** We have added the complete name/description before used abbreviations.

**Q:** How the movement of different ethnicities to metropolises does increase beta thalassemia in the population?

**Answer:** We have rephrased the sentence for better understanding. Some ethnicities in Vietnam were found with high frequency of beta-thalassemia mutated allele, therefore, as they moved and settled in metropolises, the rate of carriers and mutated allele frequency in the new population would increase.

**Q:** Some grammatical errors

**Answer:** We have made appropriate corrections of the errors.
